# A Novel 3D *In Vitro* Platform for Pre-Clinical Investigations in Drug Testing, Gene Therapy, and Immuno-oncology

**DOI:** 10.1038/s41598-019-43613-9

**Published:** 2019-05-09

**Authors:** Olivia Candini, Giulia Grisendi, Elisabetta Manuela Foppiani, Matteo Brogli, Beatrice Aramini, Valentina Masciale, Carlotta Spano, Tiziana Petrachi, Elena Veronesi, Pierfranco Conte, Giorgio Mari, Massimo Dominici

**Affiliations:** 1 Rigenerand srl, Medolla, Modena, Italy; 2 Division of Chest Surgery, Department of Medical and Surgical Sciences for Children & Adults, University-Hospital of Modena and Reggio Emilia, Modena, Italy; 3 Division of Oncology, Department of Medical and Surgical Sciences for Children & Adults, University-Hospital of Modena and Reggio Emilia, Modena, Italy; 4 Technopole of Mirandola, Mirandola, Modena Italy; 5 Medical Oncology 2, Veneto Institute of Oncology IOV, Istituto di Ricovero e Cura a Carattere Scientifico (IRCCS), Padova, Italy; 6 Department of Surgery, Oncology and Gastroenterology, University of Padova, Padova, Italy

**Keywords:** Cancer models, Cancer

## Abstract

Tumors develop within complex cell-to-cell interactions, with accessory cells playing a relevant role starting in the early phases of cancer progression. This event occurs in a three-dimensional (3D) environment, which to date, has been difficult to reproduce *in vitro* due to its complexity. While bi-dimensional cultures have generated substantial data, there is a progressive awareness that 3D culture strategies may rapidly increase the understanding of tumor development and be used in anti-cancer compound screening and for predicting response to new drugs utilizing personalized approaches. However, simple systems capable of rapidly rebuilding cancer tissues *ex-vivo* in 3D are needed and could be used for a variety of applications. Therefore, we developed a flat, handheld and versatile 3D cell culture bioreactor that can be loaded with tumor and/or normal cells in combination which can be monitored using a variety of read-outs. This biocompatible device sustained 3D growth of tumor cell lines representative of various cancers, such as pancreatic and breast adenocarcinoma, sarcoma, and glioblastoma. The cells repopulated the thin matrix which was completely separated from the outer space by two gas-permeable membranes and was monitored in real-time using both microscopy and luminometry, even after transportation. The device was tested in 3D cytotoxicity assays to investigate the anti-cancer potential of chemotherapy, biologic agents, and cell-based therapy in co-cultures. The addition of luciferase in target cancer cells is suitable for comparative studies that may also involve parallel *in vivo* investigations. Notably, the system was challenged using primary tumor cells harvested from lung cancer patients as an innovative predictive functional assay for cancer responsiveness to checkpoint inhibitors, such as nivolumab. This bioreactor has several novel features in the 3D-culture field of research, representing a valid tool useful for cancer investigations, drug screenings, and other toxicology approaches.

## Introduction

The interest in three-dimensional (3D) cell culture models is constantly increasing due to the recognized advantages of 3D *in vitro* growth compared with the traditional two-dimensional (2D) monolayer cell cultures^1,2^.

3D models can mimic *in vivo* cellular behavior providing more physiologically relevant information on cell growth and responses to a variety of chemical, physical, and immunological stimuli^3,4^. The pharmaceutical industry is one of the most relevant field requiring breakthrough 3D technologies to fill possible gaps between *in silico* hypotheses/*in vitro* results and the *in vivo* settings^4^. Although high throughput technologies offer the possibility to screen large amounts of putative drugs, the research and development of physiologically relevant compounds still require *in vivo* testing before progressing towards clinical studies^5^.

In addition to increasing ethical concerns, animal studies may be cumbersome and not always reproduce human diseases, thus, alternative or at least complementary pre-clinical tools are needed^6,7^. This is particularly true for the research on new anti-cancer compounds requiring complex interactions between different cell types such as cancer and immune cells that may be extremely difficult to reproduce during animal investigations^8^. Furthermore, alternative tests to rapidly predict anti-cancer actions are needed to reduce the relevant attrition rate during drug development^9–11^.

3D tumor cultures are a promising tool for *in vitro* rebuilding the *in vivo* behavior of cancer cells for the development and validation of anti-tumor therapies. Various 3D cell culture technologies have been created to better represent *in vivo* biology, however, advantages and limitations exist^12–14^. Therefore, we developed a novel 3D culture system as a tool that contributes to bridge *in vitro* and *in vivo* studies. This tool, named VITVO, is a small flat bioreactor that can rapidly recreate *in vitro* an *in vivo*-like environment to host a variety of normal and pathological cells in a 3D manner within a completely closed environment. Herein, the use of VITVO in several assays focusing on oncology is reported and the efficacy of chemotherapy, biologics, and cell-based anti-cancer agents was tested by comparing VITVO with an *in vivo* preclinical xenotransplant model. The flexibility offered by this novel platform within the different approaches indicates the potential of VITVO as an innovative 3D *in vitro* model for preclinical testing.

## Results

### The 3D bioreactor: structure and potential

**Figure 1:**
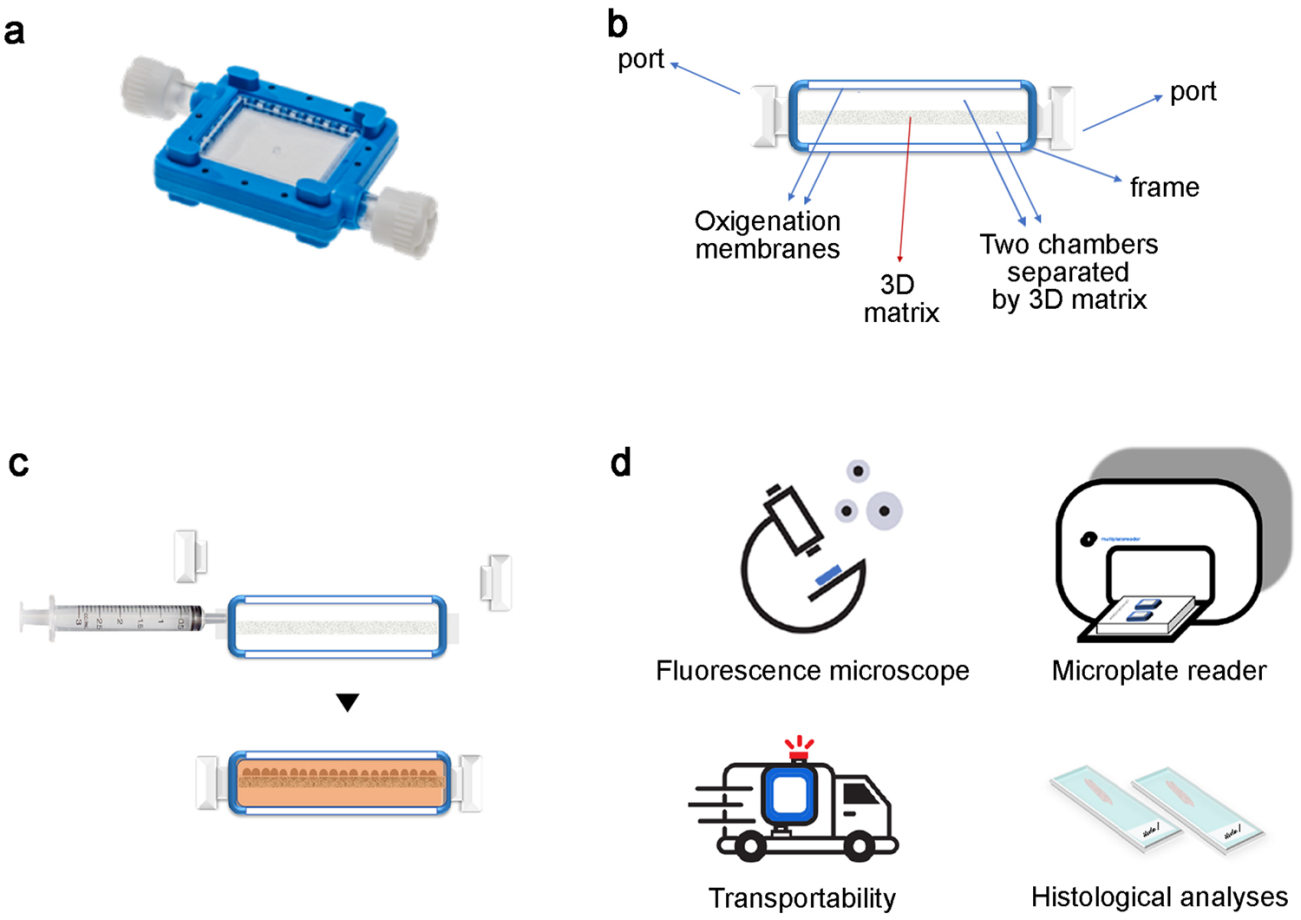
VITVO technology and potentials. (**a**) Picture of the VITVO platform. (**b**) VITVO cartoon section showing structure and parts. (**c**) VITVO loading of cell suspension with a syringe allows colonization of the 3D matrix inner core. (**d**) Cells can be directly visualized in VITVO under a fluorescence microscope; cellular viability and growth can be monitored using luminescence or fluorescence with a plate reader instrument; VITVO system is closed, thus the device is easily transported and can be shipped; methacrylate embedding of 3D tissue rebuilt in VITVO allows histological studies on tissue slices.

VITVO is a small and portable bioreactor developed to recreate a 3D tissue-like structure in a closed system that is easy to study and monitor over time (Fig. 1a). The bioreactor is formed by a perimetral frame continuous with two optical transparent oxygenation membranes which allow for gas exchange and visibility; the 3D inner core is a fiber-based matrix composed of an inert and biocompatible synthetic polyester. The matrix has a thickness of 400 μm and its empty volume represents approximately 90% of the entire volume. Internally, VITVO consists of two chambers separated only by the 3D matrix (Fig. 1b). Each chamber has a port acting as an inlet or outlet depending on the media flow; the liquid enters the first chamber and then fills the second chamber passing through the 3D structure. Thus, the 3D matrix acts as a filter allowing cell retainment and the subsequent colonization of both fibers and the empty volume between (Fig. 1c). Cell suspension can be injected in VITVO with a syringe connected to the inlet port (Video). After loading, cells may be directly viewed under a fluorescence microscope and cellular growth can be monitored and quantified using either luminescence or fluorescence with a plate reader. In addition, due to the closed system design, the device can be shipped from the loading laboratory to the read-out laboratory where 3D cultures can be monitored in real-time and processed for histological analyses (Fig. 1d and Supplementary Fig. 1).

### VITVO is biocompatible and allows real-time monitoring of 3D tumor cell growth

**Figure 2:**
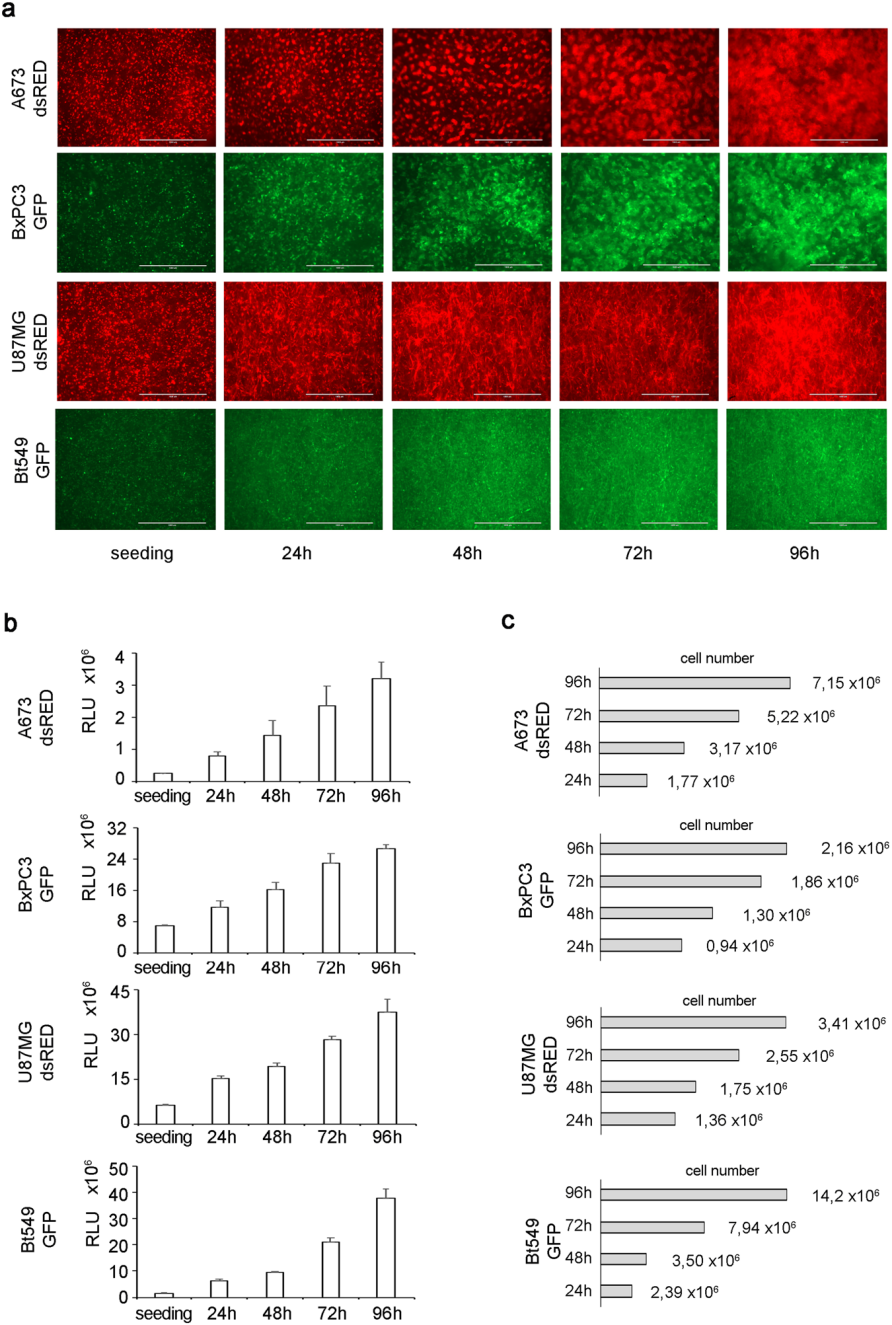
Cell growth in VITVO. (**a**) In VITVO tumor growth monitoring under a fluorescence microscope. Scale bar 1,000 μm. (**b**) In VITVO cell viability monitoring using Real Time GLO (Promega). Real Time GLO was added at 1X concentration at seeding, at 24 h and 48 h for A673 dsRED, U87MG dsRED and Bt549 GFP, while it was added at 2X concentration for the 72 h and 96 h time points. For BxPC3 GFP, Real Time GLO was added 1X concentration at seeding and at 24 h, and 2X concentration at 48 h/72 h/96 h. All measurements were performed in triplicate and data are expressed as means ± standard deviation (SD) (**c**) Estimation of tumor cells number in VITVO based on relative light units (RLU; cell number = measured RLU*cell number at seeding/RLU at seeding).

Several tumor cell lines derived from different histotypes were introduced to assess VITVO biocompatibility and its capacity to support high-density 3D cell growth. To directly visualize cells using fluorescence microscopy, the cell lines were gene-modified to stably express two fluorescent proteins (dsRED: red and GFP: green). Ewing sarcoma (A673 dsRED^+^), glioblastoma (U87MG dsRED^+^), breast cancer (Bt549 GFP^+^), and pancreatic (BxPC3 GFP^+^) tumor cell lines were then loaded in VITVO and their growth was monitored for up to 96 hours after seeding (Fig. 2). The progressive 3D matrix colonization by labeled proliferating cells was directly monitored under a fluorescence microscope (Fig. 2a). The 3D matrix alone with culture media did not have any autofluorescence in both GFP and RFP channels (not shown).

To further confirm the data and generate quantitative measures, the number of living cells inside VITVO for each cell line was then evaluated using the Real Time-Glo MT cell viability assay with a luminometer, as an additional applicable read-out in combination with the microscopy.

The addition of Real Time-Glo reagent in culture media produce a bioluminescence signal proportional to the number of viable cells. Cells loaded in VITVO and incubated with Real Time-Glo generated a gradual increase in bioluminescence signal, thus indicating viable cells progressively proliferate and colonize the 3D matrix (Fig. 2b).

Using this assay, cell growth could be directly monitored over time inside the system by measuring the relative light units (RLU). Considering the number of cells (5.6 × 10^5^ cells/device) initially loaded into the VITVO system and using RLU values at each time point, estimating the number of cells inside the system up to 96 hours was possible (Fig. 2c).

These findings indicate VITVO is biocompatible and can sustain a 3D cell growth in a variety of cancer types which can be monitored using both microscopy and luminometry.

### VITVO supports 3D cytotoxicity assay

After observing the capacity of VITVO to provide a 3D support for cancer cell line proliferation, the use of the system as a tool to monitor the impact of a known chemotherapy agent against a selected cell line was investigated.

**Figure 3:**
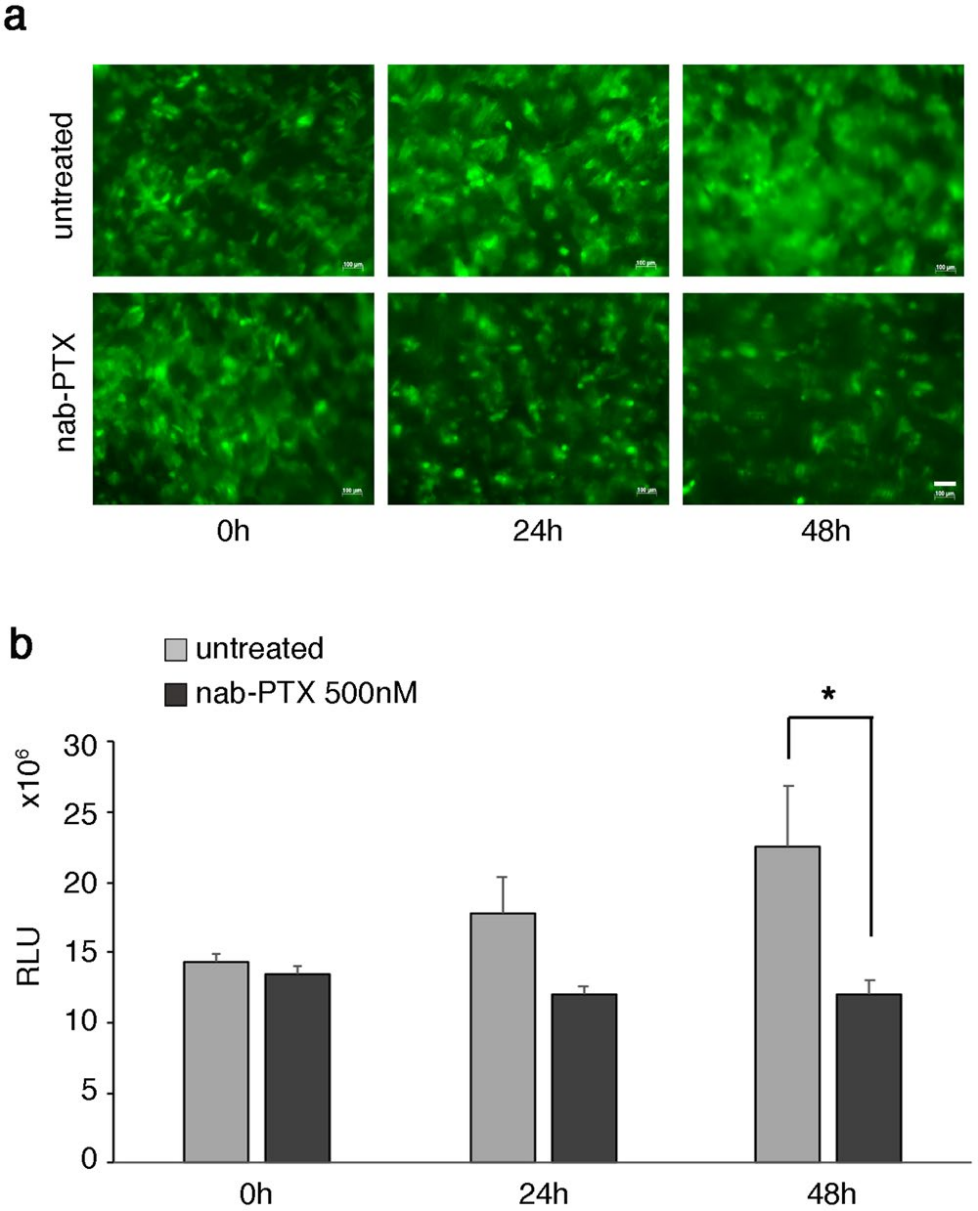
In VITVO efficacy evaluation of a chemotherapy agent. (**a**) In VITVO visualization of GFP^+^ BxPC3 under a fluorescence microscope. Scale bar 100 μm. (**b**) In VITVO growth monitoring of tumor cells after treatment with 500 nM nab-PTX using Real Time GLO viability assay. Untreated cells were used as control. All measurements were performed in triplicate and data expressed as means ± SD, *P = 0.015 (*t*-test).

Thus, the pancreatic tumor cell line BxPC3 expressing GFP was loaded in VITVO and 24 hours after the seeding, cells were treated with nab-paclitaxel (nab-PTX) (Fig. 3). Tumor cell viability was regularly monitored using both microscopy and Real Time-Glo assay. A reduction of GFP^+^ cell density was clearly observed under fluorescence microscope 24 hours after adding the drug. In treated samples, the cytostatic action of nab-PTX markedly affected the tumor cell growth compared with untreated controls where BxPC3 progressively colonized VITVO (Fig. 3a).

At 48 hours, this observation was further confirmed with a significant reduction of the green signal. Cell growth was additionally monitored using the cell viability assay and after 48 hours of nab-PTX treatment, a significative reduction of cell viability (RLU) was observed in treated samples compared with untreated controls (Fig. 3b), confirming the data of the microscopic evaluations and suggesting that VITVO is a suitable tool for testing cytotoxicity at different time points and with distinct read-outs.

### VITVO hosts a biologic-agent driven pro-apoptotic effect visible using the luciferase assay

Starting with the cytotoxicity generated by a chemotherapy agent in VITVO, the system was further tested using a pro-apoptotic biologic agent, the soluble form of TNF-related apoptosis-inducing ligand (sTRAIL)^15^. sTRAIL induces tumor cell death by interacting with specific receptors; this event triggers intracellular signals that ultimately activate caspases which induce apoptosis. In addition, decoy receptors can interfere with the mechanism of action^16^. Moreover, sTRAIL has a relevant molecular weight (at least 36 KDa) significantly higher than a chemical compound, possibly interfering with its biodistribution and penetration in a 3D structure. Considering the complexity of the mechanism that induces apoptosis and accounting for the 3D structure, sTRAIL in VITVO was tested against an Ewing sarcoma cell line with *in vitro* and *in vivo* TRAIL sensitivity^17^. Two different bioluminescent-based approaches were compared; in addition to the Real Time-Glo assay applied to gene-engineered dsRED positive A673 cell line (dsRED^+^ A673), a second dsRED negative A673 (dsRED^−^ A673) cell line variant expressing luciferase (Luc^+^ A673), which is typically used in *in vivo* xenotransplants, was generated.

**Figure 4:**
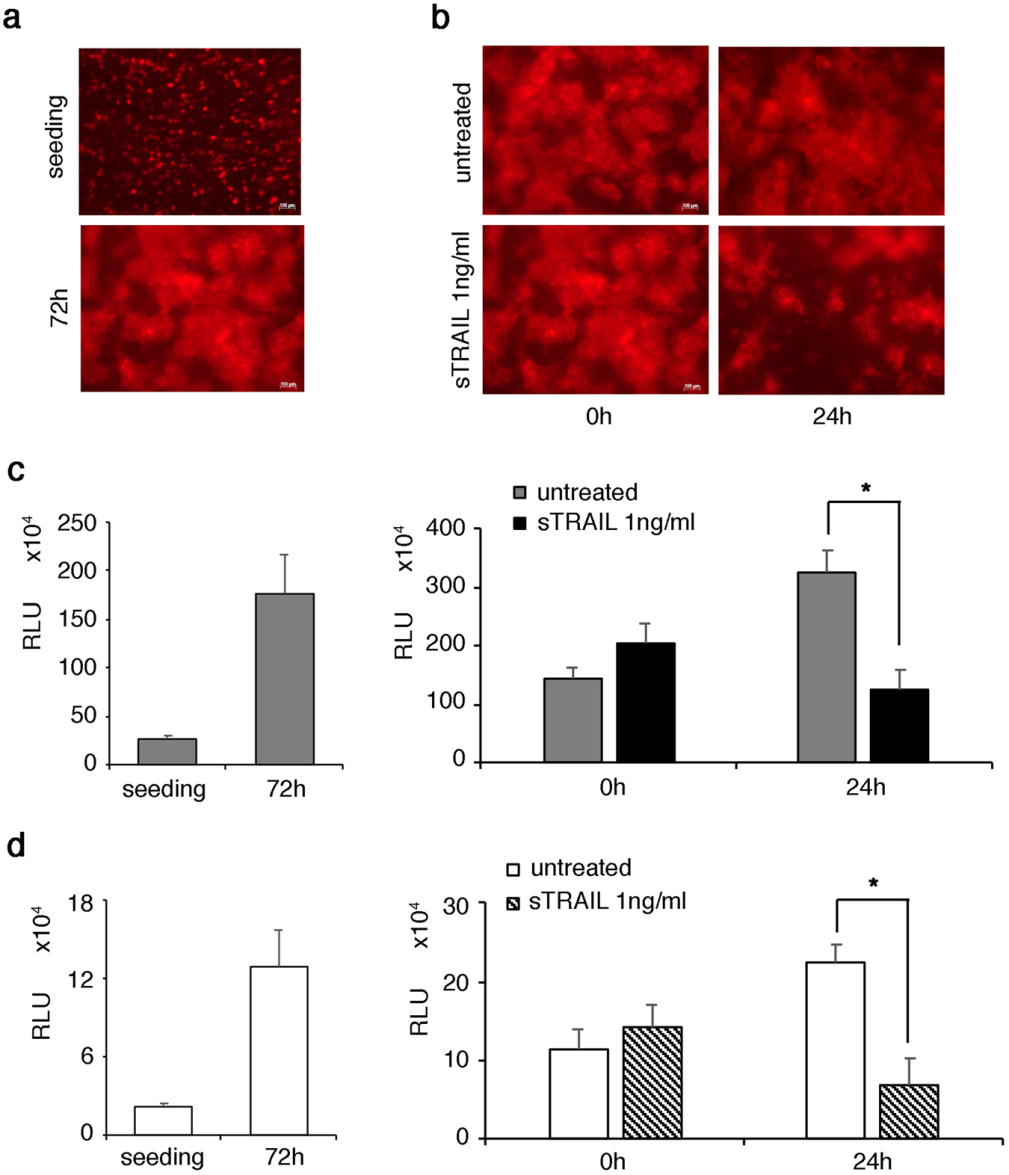
In VITVO evaluation of sTRAIL pro-apoptotic action on Ewing sarcoma using two different luminometric approaches. (**a**) In VITVO dsRED^+^ A673 visualization using fluorescence microscopy at seeding and after 72 hours. Scale bar 100 μm. (**b**) In VITVO monitoring of sTRAIL action against tumor using microscope visualization of fluorescent cells during treatment (0 hour and 24 hours). (**c**) In VITVO measurement of dsRED^+^ A673 viability based on RealTime-Glo. Cell growth was monitored for 72 hours (left panel), then sTRAIL was added in VITVO and cell viability was measured after 24 hours based on luminescence (right panel). Untreated dsRED^+^ A673 cells were used as control. All measurements were performed in triplicate and data are expressed as means ± SD *P = 0.003. (**d**) In VITVO measurement of Luc^+^ A673 viability by the addition of luciferin. Cell growth was monitored for 72 hours (left panel), then sTRAIL was added in VITVO and cell viability was measured after 24 hours based on luminescence (right panel). Untreated Luc^+^ A673 cells were used as control. All measurements were performed in triplicate and data are expressed as means ± SD, *P = 0.002.

DsRed^+^ A673 or Luc^+^ A673 cells were then loaded in VITVO and grown for 72 hours to repopulate the 3D matrix and obtain a consistent tumor bulk. dsRED^+^ A673 growth was monitored using fluorescence microscopy (Fig. 4a), confirming that VITVO can support sarcoma 3D proliferation. When sTRAIL treatment was started, the pro-apoptotic effect was clearly visible with a reduction of red fluorescence at 24 hours, indicating the relevant anti-sarcoma effect was triggered by sTRAIL (Fig. 4b). The data were then confirmed using Real Time-Glo assay which showed the rapid VITVO repopulating activity of dsRED^+^ A673 cells (Fig. 4c, left panel), as well as the capacity of sTRAIL to significantly reduce the number of living cells in 24 hours (Fig. 4c, right panel). Simultaneously, the performance of Luc^+^ A673 cells was evaluated by adding the luciferin substrate into the culture and measuring the RLU caused by luciferase activity. The luciferase assay confirmed VITVO can host the rapidly growing Ewing sarcoma cell line (Fig. 4d, left panel), further demonstrating the assay can prove cell death with comparable results between the two luminometric readouts (Fig. 4d, right panel).

Thus, VITVO can be used to explore the anti-cancer potential of biologic agents in 3D and the addition of luciferase in target cells is suitable for comparative studies that may also involve parallel *in vivo* investigations.

### VITVO can be loaded with primary lung cancer cells targeted by nivolumab to rapidly identify an immune-based anti-tumor response

Based on the results obtained using VITVO against tumor cell lines, whether the system could also host human primary tumor cells together with their accessory cells was investigate, as prerequisite before introducing the device as a predictive tool in oncology. Specifically, in addition to histological assessment of PD1-PDL1, the focus was on immuno-oncology agents, since rapid functional tests that can predict the efficacy of checkpoint inhibitors are lacking^18^.

**Figure 5:**
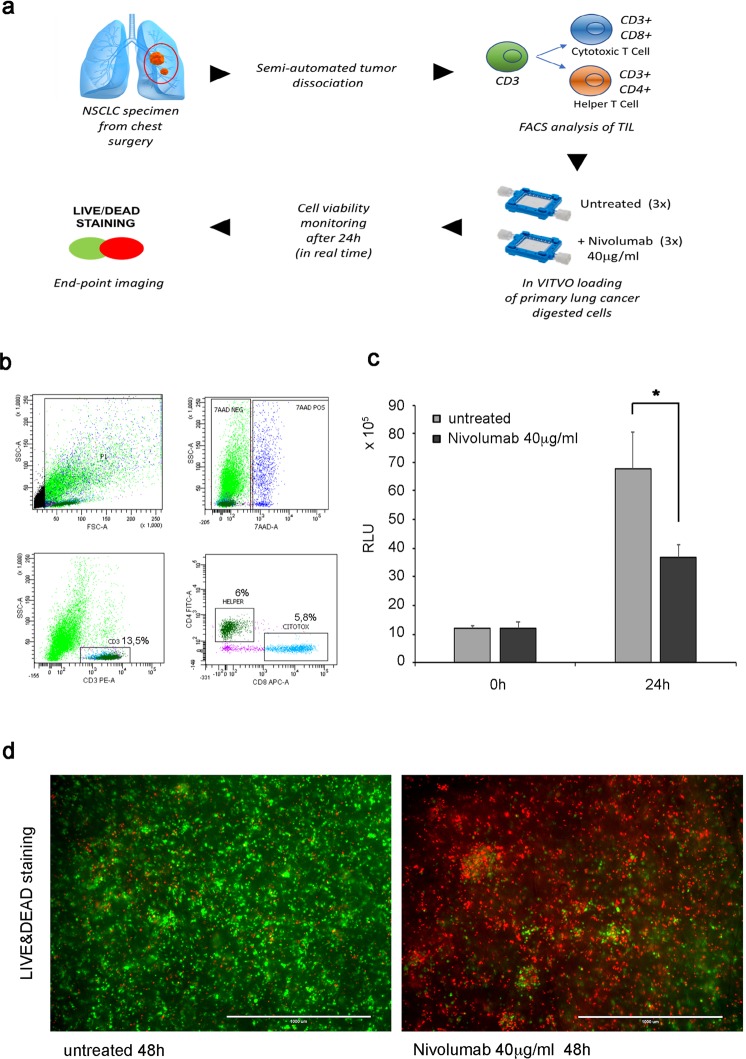
In VITVO testing of the effect of nivolumab on lung primary tumor. (**a**) Experimental scheme. (**b**) Immunostaining of unfractionated tumor cells dissociated from lung cancer specimen with anti-CD3, anti-CD4, and anti-CD8 to quantify the percentage of immune cells (CD3^+^) and the cell subpopulations of lymphocyte T helper (CD4^+^) and lymphocyte T cytotoxic (CD8^+^). (**c**) In VITVO cell viability evaluation based on RealTime-Glo at seeding and after 24 hours of treatment with nivolumab. Real Time GLO was used at a 1X final concentration for both measurements. Untreated samples were used as control. All measurements were performed in triplicate and data are expressed as means ± SD, *P = 0.01. (**d**) In VITVO live and dead staining after nivolumab treatment (48 hours) to visualize live (green) and dead (red) cells. Representative images of untreated sample (left pic) and treated sample (right pic) are shown. Scale bar 1,000 μm.

Therefore, the use of VITVO for evaluating the efficacy of nivolumab in promoting anti-tumor immunity of tumor-infiltrating lymphocytes (TILs) in non-small-cell lung cancer (NSCLC) was investigated following the experimental scheme shown in Fig. 5a. After obtaining approved informed consent, NSCLC specimens were obtained from chest surgery patients and tumor cells were dissociated using a semi-automated combined mechanical/enzymatic process. Digested cells were first evaluated using fluorescence-activated cell sorting (FACS) to identify and quantify TILs (Fig. 5b) and unfractionated cells (2 × 10^6^) were loaded in VITVO with 40 μg/mL of nivolumab; untreated cells were used as control. Real Time-Glo was then added to monitor cell viability measured at seeding and after 24 hours. As shown in Fig. 5c, the viability of treated samples was dramatically compromised compared with the untreated samples, suggesting a possible trigger of anti-tumor activity of cytotoxic TILs by nivolumab.

In addition to the Real Time-Glo to visualize tumor cell death in the matrix using microscopy, another assay that can discriminate between living (green) and dying cells (red; Fig. 5d) was used. The fluorescence-based Live/Dead assay was performed over a longer time period (48 hours) showing a significant increase of red-colored cells in nivolumab-treated samples and confirmed the results obtained using the bioluminescence approach. Collectively, these results indicate a proof of concept in which VITVO can host primary tumor cells harvested from a tumor specimen to be rapidly (<48 hours) introduced in a functional assay for cancer responsiveness to checkpoint inhibitors.

### In VITVO dose finding study for a gene therapy-based approach

During the development of cell-based approaches in cancer, a major limitation is identification of the proper cell dose that should be associated with the desired therapeutic outcome (https://www.fda.gov/downloads/BiologicsBloodVaccines/GuidanceComplianceRegulatoryInformation/Guidances/CellularandGeneTherapy/UCM564952.pdf).

The progressive introduction of cancer cell therapies into the clinic, in addition to animal models that may be limited in hosting human cancers^18^, should require *in vitro* tools that can mimic the clinical setting in which cell growth can influence molecular biomarkers and antigenic profiles^19–22^. Therefore, whether a 3D system such as VITVO may have value in simultaneously hosting target and effector cells and identifying the effective dose compared with the dose determined in an animal study was investigated. Thus, luciferase-positive pancreatic adenocarcinoma cells (Luc^+^ BxPC-3) were loaded in VITVO repopulating the 3D inner core; after 24 hours, adipose-derived mesenchymal stromal cells (AD-MSCs), genetically modified to secrete soluble TRAIL (MSC-sTRAIL)^23^, were injected in VITVO at different effector:target ratios (E:T 1:10 and 1:30). Then, luciferin was added to the system to evaluate cell viability at different time points by measuring RLU.

**Figure 6:**
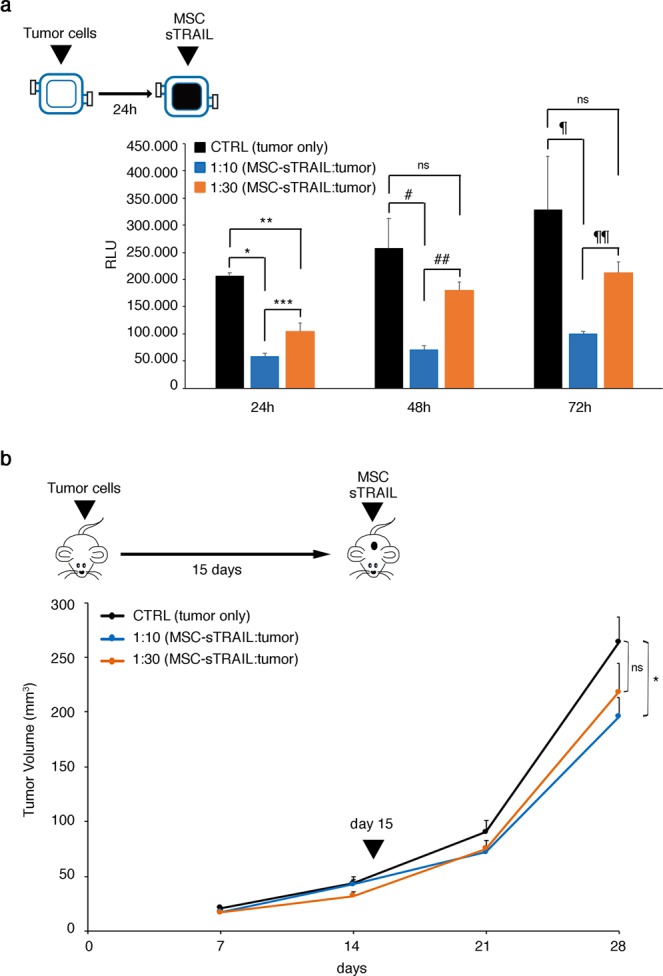
In VITVO dose finding for cell-based therapy. (**a**) In VITVO cell viability of Luc^+^BxPC3 was evaluated by the addition of luciferin after treatment with MSC-sTRAIL at different effector:tumor ratios (E:T 1:10 and 1:30) at 24-, 48-, and 72-hour time points. Untreated Luc^+^ BxPC3 cells were used as control. All measurements were performed in triplicate and data are expressed as means ± SD. *P = 0.000003, **P = 0.0004, ***P = 0.007, ^#^P = 0.004, ^##^P = 0.004, ^¶^P = 0,015, ^¶¶^P = 0.0006, based on ANOVA and multiple comparisons. (**b**) *In vivo* xenotransplant of BxPC3 and evaluation of tumor inhibition by a single subcutaneous intratumoral injection of MSC-sTRAIL at different effector:tumor ratios (E:T 1:10 and 1:30). Untreated mice were used as control. Values are expressed as means ± SEM; *P = 0,04 based on ANOVA and multiple comparisons.

After 24 hours of 3D co-culture with MSC-sTRAIL and Luc^+^ BxPC-3, both doses were found effective against pancreatic adenocarcinoma cells. However, at a longer observation time point (48 hours and 72 hours), the lowest ratio (1:30) of cells did not control tumor growth while the highest ratio (1:10) maintained a statistically significant reduction in tumor bulk compared with control (Fig. 6a).

To assess the VITVO potential in this context, the obtained VITVO results were compared with those generated by an *in vivo* subcutaneous xenotransplant model (Fig. 6b). After subcutaneous injection of BxPC-3 (2 × 10^6^), mice were divided into three groups (n = 6 mice per group): control (no treatment), E:T 1:10 (E: 4 × 10^6^), and E:T 1:30 (E: 1.3 × 10^6^). A single intratumoral injection of AD-MSC sTRAIL in subcutaneous pancreatic tumors was administered 15 days after BxPC-3 subcutaneous implantation. Tumor mass growth was monitored during the experiment, and after 28 days, a statistically significative reduction in tumor volume compared with untreated group was observed only in the E:T 1:10 group. This result is in agreement with the in VITVO results and indicates dose-finding studies using in VITVO as a complementary tool for anti-cancer drug discovery can be performed.

## Discussion

Cell culture models are fundamental instruments for basic to translational research, in particular for cancer^24,25^. Although *in vitro* conditions do not exactly replicate *in vivo* situations, their role remains important^3^. Conversely, while *in vivo* data are critical in drug discovery, a comprehensive survey of drug screening tests has shown that approximately half of toxicological data derived from experiments with rodents does not correlate with the results from human trials^26^. Moreover, pre-clinical toxicity studies with multiple animal species may be poorly predictive, and in several cases, drugs have failed during clinical trials in humans due to unexpected adverse toxicities^27,28^. Therefore, development of new *in vitro* models that can mimic the *in vivo* complexity and in combination with reliable animal models are needed to overcome these issues.

3D tissue cultures are fast emerging tools to better investigate cell biology and improve the clinical relevance of culturing cells outside a living body^29,30^. Based on bioreactor technology and considering these unmet needs, we developed VITVO as an innovative 3D tool providing a tissue-sized platform where cells can recreate their environment with a higher level of complexity and in a simple manner.

Focusing on experimental oncology, the VITVO flexibility allowed testing of several approaches with a variety of read-outs. Conventional assays developed to study biological function and for testing anti-tumor pharmacodynamics are generally performed using 2D monolayer cultures; however, limits in predictability of the *in vivo* outcomes are well documented^1,2^. Therefore, 3D cancer cell cultures are expected to better mimic *in vivo* microenvironments, providing an improved understanding of their morphological and functional peculiarities^21,22^.

The miniaturization of both 2D and 3D cultures facilitated high throughput screening (HTS) technology, allowing the possibility to screen a large number of compounds evaluating the efficacy of a single endpoint^9^. However, this approach may not be completely predictive of the compound response *in vivo*, thus generating a high attrition rate in downstream drug development pipelines^31^. The gap between *in vitro* and *in vivo* may be bridged by creating more reliable assays for HTS or conceiving breakthrough technology based on low throughput screening (LTS) that can generate high value data with an increased level of prediction. In miniaturized 3D system, such as spheroids, the floating cells form self-assembly aggregates in which some zones are not equally exposed to the environment changes (nutrients, gas and waste), impacting in the proper formation/performance of the tissue. This is particularly true for larger spheroids commonly presenting a concentrically layered structure with an internal apoptotic/necrotic core surrounded by a viable layer of quiescent cells and an external edge of proliferating cells. This situation may represent a limitation that often clashes with the possibility to obtain uniform structures able to mimic complexity and ensure reproducibility^32^. VITVO is a valuable tool to host a higher number of cells recreating a critical tumor mass with the possibility to generate an increased level of complexity by co-culturing different cell types and better mimic an *in vivo* microenvironment within a defined morphometry due to the shape of the inner core of the 3D system. As reported for other 3D cultures on scaffold^32,33^, VITVO is limited in the direct measurement of tumor size reduction after treatment, however it assesses cell viability in comparison to controls similarly to what is done when luciferase assay is performed *in vivo*. In that case, even if tumor size and residual cell number are not directly measured, the number of photons correlates with the reduction of tumor size and viable cells. Being aware of this limitation and in the attempt to overcome it, VITVO can be processed for histology where tumor mass can be also visualized for more direct histomorphological measurements (Supplementary Fig. 1).

Therefore, the system can be used in LTS strategies and the platform was validated using different types of cancer cells including mesenchymal, epithelial, and neuroectodermal tumors. Based on the data, the device can host various cell types that can be loaded into the device, repopulate the matrix, and survive in the closed environment due to the gas permeable membranes. Cell cultures were usually performed at 37 °C with 5% CO_2_, however, growing tumor cells in VITVO outside the incubator at 37 °C (for at least 48 hours) was achieved (Supplementary Fig. 2). In addition, the possibility to load a mix of different cells types originating either from freshly digested tumor samples or established cells lines (i.e. MSC and tumors) was demonstrated. Consequently, potential applications in which VITVO can be used to study the interactions between tumors and the different cells in their microenvironments (lymphocyte-subsets, myeloid derived cells, stroma, and endothelium), without limiting the number and type of cells, can be developed. Importantly, the flat nature of the system allows the monitoring of 3D matrix colonization and interaction due to cell labeling visible using microscopy and plate readers utilizing both fluorescence and luminescence. Finally, the structure enables the specific cancer niches to remain stable in the 3D matrix, allowing to follow them using microscopy without the risk of movement that could impair identification during the experimental period.

All these factors are relevant for better understanding the dynamic interactions of a tumor and rebuilding its environment in VITVO for metabolic, immuno-oncology and basic genomic investigations. By sampling the media, soluble factors released during interactions can be tested and DNA/RNA can be obtained from the cells repopulating the matrix using commercially available kits directly within the system for immediate use or storage (Supplementary Fig. 3).

The simplicity of use and the adaptability to the common laboratory equipment may allow a rapid transfer of the technology into both academic and pharmaceutical research and development fields. Due to the closed system design, VITVO additionally offers the advantage of transportability; the tumor sample can be loaded at the clinical facility and then shipped to a remote laboratory for the necessary tests.

All these values contribute to build a 3 V concept: *in vitro*, in VITVO and *in vivo*. *In vitro* HTS provides the identification of new drug candidates; in VITVO testing can reveal pharmacodynamics and efficacy dose ranges of these compounds using primary and/or established cell line in 3D, to then proceed *in vivo* with more relevant information, ultimately reducing the number of animal investigations with a significant lowering in the attrition rate of drug development.

An additional relevant application of the VITVO system is in the field of immuno-oncology having value both in drug development and clinical settings, where rapid functional tests allowing prediction of patient response are still lacking^9^. In the present study, freshly isolated cells collected from lung cancer patients and loaded in VITVO without the addition of effector cells, elicited the TIL activation caused by nivolumab against tumor cells. To the best of our knowledge, this process has not been previously described and presumably, the 3D growth generates favorable cell-to-cell interactions playing a key role in obtaining a closer relationship between target and effectors, as observed in patients. Subsequently, an anti-tumor response is generated due to anti-PD1 antibodies and for better tumor antigen expression caused by 3D cell growth, as previously reported^21,22^.

In conclusion, a novel device for 3D cell culture was introduced in the present study. The simplicity of use and readout flexibility of VITVO allows a variety of pre-clinical investigations in oncology, with a possible relevant impact in other areas, such as toxicology studies using hepatocytes and for other more complex toxicology assays using inducible progenitors.

## Methods

### VITVO loading and 3D cell culture establishment

VITVO was first primed with media alone to ensure the complete wetting of 3D matrix. Next, 5.6 × 10^5^ cells were suspended in 1.4 mL of culture media and injected into the system with a 5 mL syringe (Becton Dickinson and Co, Franklin Lakes, NJ, USA). For each cell type, culture media were changed every 24 hours.

### Tumor cell lines

The following tumor cell lines were purchased from ATCC (LGC Standards srl, Milan, Italy) and genetically modified by viral infection to express fluorescent protein: Retroviral vectors MSCV-GFP and MSCV-dsRED were transiently transfected into 293 T cells together with a mixture of helper plasmid according to the jetPEI protocol (Cell Signaling Technology Inc., Danvers, MA, USA). After 48 hours, conditioned media containing retroviral particles were collected to stably transduce FLYRD18 packaging cell line for the continuous production of viral particles. FLYRD18-conditioned media were used to perform 3 hits of infection on tumor cell lines (6 hours of incubation/day).

Ewing sarcoma A673 was modified to express dsRED protein, pancreatic adenocarcinoma BxPC3 modified to express GFP protein, glioblastoma U87MG modified to express dsRED protein, and breast cancer Bt549 modified to express GFP protein.

Luciferase-positive BxPC3 was obtained using lentiviral particles purchased from Perkin Elmer (Waltham, MA, USA).

A673 and U87MG were cultivated in Iscove (Euroclone, Padmington, UK), and BxPC3 and Bt549 were seeded in RPMI-1640 (Thermo Fisher Scientific, Waltham, MA, USA).

Media were supplemented with 10% fetal bovine serum (FBS, PAA, Pasching, Austria), 1% glutamine (200 mM), and 1% penicillin‐streptomycin (10^4^ UI/mL and 10 mg/mL; both from Euroclone).

### In VITVO observation using fluorescence microscopy

Cells in VITVO were directly visualized with an EVOS FL auto (Thermo Fisher Scientific) using GFP and RFP LED cubes and/or an Axiovert 200 M fluorescence inverted microscope (Zeiss, Oberkochen, Germany).

### In VITVO growth monitoring using luminescence

Cell growth evaluation was performed using the Real Time-Glo MT cell viability assay (Promega, Madison, WI, USA) with a protocol specifically developed for VITVO. This protocol was designed to ensure sufficient quantity of reagent considering the enlarged model compared with the miniaturized system. Real Time-Glo was added to the cells immediately before the cell loading in VITVO at a final 1X concentration. After incubation, Relative Light Units (RLU) were measured using a luminometer (GloMax Discover System, Promega). The reagent was re-added at each change of media (1X or 2X final concentration), depending on the cell type and considering the size and growth rate. 2X concentration allows having enough quantity of substrate for high number of cells in culture, however the emitted signal (RLU) remains directly proportional to the number of viable cells. Thus, the substrate has not been considered a limiting factor even at lower concentration (Supplementary Fig. 4). After incubation, RLU were remeasured using the luminometer. Since RLU value is directly proportional to the number of viable cells and knowing the number of cells at seeding and the corresponding RLU value, the number of cells in VITVO was estimated using the measured RLU at each time point.

RLU were measured directly from VITVO using a tray and setting the instrument (GloMax Discover System, Promega) to read the two central wells of a 6 multi-well plate. Viability of luciferase-positive cells loaded in VITVO was evaluated by adding the substrate luciferin (Promega) into culture and measuring RLU.

### Drugs and treatments in VITVO

For testing Nab-paclitaxel (Abraxane, Celgene, Milan, Italy), sTRAIL, and MSC-sTRAIL were used at the specified concentrations and added in VITVO within the culture media as a single dose.

### Primary cells from human lung cancer

The study was approved by the Ethical and Institutional Review Board of the University Hospital of Modena and was performed in accordance with the relevant guidelines and regulations. Tumor specimens were obtained after obtaining signed informed consent from patients who underwent surgery at the Chest Surgery Division (University Hospital of Modena). Tumor tissues were dissociated into single cells using the Human Tumor Dissociation Kit (MACS, Miltenyi Biotec, Bergisch Gladbach, Germany) with GentleMACS Octo Dissociators and heater protocols (Miltenyi Biotec). Immunophenotyping of cells recovered after tumor tissue digestion was performed. Cells were counted, seeded in FACS analysis polypropylene tubes (1 × 10^6^/tube; VWR, Milan, Italy) and incubated in blocking buffer (100 μL each 1 × 10^6^ MSC) containing Dulbecco’ s modified Eagle medium (DMEM), 10% FBS, 0.1 M sodium azide, and 66.6 mg/mL human immunoglobulin G (Sigma, Steinheim, Germany) for 20 minutes on ice. Cells were subsequently stained for 30 minutes on ice with primary antibodies in PBS with 0.1% bovine serum albumin (BSA; Sigma) and analyzed using a FACS ARIA III (BD Bioscience, Franklin Lakes, NJ, USA). The following monoclonal antibodies were used: CD3-PE, CD-8-APC, CD4-FITC; 7-amino-actinomycin-D (7AAD)-staining (ViaProbe; BD Biosciences; 20 μL/1 × 10^6^ cells, according to the manufacturer’ s instructions) and evaluated using flow cytometry to detect apoptosis.

Primary cells were plated in RPMI-1640 (Thermo Fisher Scientific), supplemented with 10% FBS (PAA), 1% glutamine (200 mM), and 1% penicillin‐streptomycin (10^4^ UI/mL and 10 mg/mL; both from Euroclone).

### *In vivo* dose finding

All animal experiments were approved by the Animal Ethics Committee of Modena and all methods were performed in accordance with the relevant guidelines and regulations. Tumor implantation and cell delivery were performed as described in the Results section.

NOD.CB17-Prkdc^scid^/NCrCrl mice were purchased from Charles River (Wilmington, MA, USA).

### Statistics

Data were analyzed using Microsoft Excel 2016 (Microsoft Corporation) and expressed as mean values ± standard deviation (SD) or ± standard error of the mean (SEM). Unpaired two-tailed Student’s *t*-test was used and a p-value ≤ 0.05 was considered statistically significant. ANOVA test was performed using Prism software (GraphPad 8 Software Inc., La Jolla, CA, USA).

## Supplementary information

VITVO video

Supplementary Information

## Data Availability

The datasets generated during and/or analyzed during the current study are available from the corresponding author upon reasonable request.
