# Author Correction: A Novel 3D *In Vitro* Platform for Pre-Clinical Investigations in Drug Testing, Gene Therapy, and Immuno-oncology

**DOI:** 10.1038/s41598-020-57846-6

**Published:** 2020-01-30

**Authors:** Olivia Candini, Giulia Grisendi, Elisabetta Manuela Foppiani, Matteo Brogli, Beatrice Aramini, Valentina Masciale, Carlotta Spano, Tiziana Petrachi, Elena Veronesi, Pierfranco Conte, Giorgio Mari, Massimo Dominici

**Affiliations:** 1 Rigenerand srl, Medolla, Modena, Italy; 2 Division of Chest Surgery, Department of Medical and Surgical Sciences for Children & Adults, University-Hospital of Modena and Reggio Emilia, Modena, Italy; 3 Division of Oncology, Department of Medical and Surgical Sciences for Children & Adults, University-Hospital of Modena and Reggio Emilia, Modena, Italy; 4 Technopole of Mirandola, Mirandola, Modena, Italy; 5 Medical Oncology 2, Veneto Institute of Oncology IOV, Istituto di Ricovero e Cura a Carattere Scientifico (IRCCS), Padova, Italy; 6 Department of Surgery, Oncology and Gastroenterology, University of Padova, Padova, Italy

Correction to: *Scientific Reports* 10.1038/s41598-019-43613-9, published online 09 May 2019

The original version of this Article contained an error in Affiliation 5, which was incorrectly given as ‘Department of Surgery, Oncology and Gastroenterology, University of Padova, Istituto Oncologico Veneto IRCCS, Padova, Italy’. The correct affiliation is listed below:

Department of Surgery, Oncology and Gastroenterology, University of Padova, Padova, Italy

In addition, the original version of this Article omitted an affiliation for Pierfranco Conte. The correct affiliations for Pierfranco Conte are listed below:

Medical Oncology 2, Veneto Institute of Oncology IOV, Istituto di Ricovero e Cura a Carattere Scientifico (IRCCS), Padova, Italy

Department of Surgery, Oncology and Gastroenterology, University of Padova, Padova, Italy

These errors have now been corrected in the HTML and PDF versions of this Article, and in the accompanying Supplementary Information.

