# Supplementary material for: A Novel 3D In Vitro Platform for Pre-Clinical Investigations in Drug Testing, Gene Therapy, and Immuno-oncology
Source: Sci Rep. 2019 May 9;9:7154. doi: 10.1038/s41598-019-43613-9 (PMC6509120; doi:10.1038/s41598-019-43613-9)

A Novel 3D *In Vitro* Platform for Pre-Clinical Investigations in Drug Testing, Gene Therapy, and Immuno-oncology

Olivia Candini*[1], Giulia Grisendi[1], Elisabetta Manuela Foppiani[1], Matteo Brogli[1]
Beatrice Aramini[2], Valentina Masciale[3], Carlotta Spano[1], Tiziana Petrachi[4],
Elena Veronesi[4], Pierfranco Conte[5,6], Giorgio Mari[1] & Massimo Dominici*[1,3]

[1]Rigenerand srl, Medolla, Modena, Italy; [2]Division of Chest Surgery, Department of Medical and Surgical Sciences for Children & Adults, University-Hospital of Modena and Reggio Emilia, Modena, Italy; [3]Division of Oncology, Department of Medical and Surgical Sciences for Children & Adults, University-Hospital of Modena and Reggio Emilia, Modena, Italy; [4]Technopole of Mirandola, Mirandola, Modena, Italy; [5] Medical Oncology 2, Veneto Institute of Oncology IOV, Istituto di Ricovero e Cura a Carattere Scientifico (IRCCS), Padova, Italy; [6]Department of Surgery, Oncology and Gastroenterology, University of Padova, Padova, Italy.

***Co-corresponding Authors:**

[1] Olivia Candini, PhD: olivia.candini@rigenerand.it

[2] Massimo Dominici, MD: massimo.dominici@unimore.it

Supplementary materials and methods

Histology. 3D matrices were fixed with 10% buffered formalin for 30 minutes, then alcoholic scale before methacrylate embedding was performed. Methacrylate-embedded matrix sections were evaluated by hematoxylin and eosin (H&E) staining (Sigma Aldrich).

RNA Extraction and cDNA Synthesis. Total cellular RNA was isolated using TRIzol (Invitrogen, Carlsbad, MN, USA). The RNA was quantified using a spectrophotometer (Beckman Coulter DU 730, Pasadena, CA, USA). First-strand complementary DNA

(cDNA) was synthesized from 2 µg total RNA using the RevertAid H minus first-strand cDNA synthesis kit (Fermentas - ThermoFisher, Waltham, Massachusetts, USA).

Quantitative Real-Time PCR. Quantitative real-time PCR (qRT-PCR) was performed using the Applied Biosystems StepOne Real-Time PCR System and the Fast SYBR Green Master Mix reagent. The qRT-PCR reaction (10 µL) consisted of 50 ng cDNA, Fast SYBR Green Master Mix (Applied Biosystems, Foster City, California, USA), and 300 nM of the forward and reverse primers; β-actin 5'-ACCTTCTACAATGAGCTGCG-3' (sense) 5'-CCTGGATAGCAACGTACATGG-3' (antisense), GAPDH 5'-ACATCGCTCAGACACCATG-3' (sense) 5'-TGTAGTTGAGGTCAATGAAGG-3' (antisense).

Supplementary legends

Supplementary Figure 1

Histology evaluation of the 3D culture. (a) After culture, the 3D matrix inner core of VITVO was removed from the device, using a surgical scalpel to cut the oxygenation membrane. The embedding of the 3D matrix in methacrylate was performed to obtain a thickness section using a microtome. **(b)** H&E staining on a section of 3D matrix colonized by A673 cells. The staining shows cell penetration through the thickness of the 3D matrix (146 µm out of the 400 µm available). Black arrows indicate some cut fibers residues. Scale bars 100 µm (left panel) and 50 µm (right panel).

Supplementary Figure 2

In VITVO cell viability outside the incubator (a) In VITVO visualization by fluorescence microscope of dsRED+ A673, cultured for 24 hours and 48 hours outside the incubator at 37°C. Scale bar 100 µm. **(b)** In VITVO cell viability monitoring by Real Time-Glo

(Promega) and estimation of A673 dsRED+ cells number in VITVO by RLU, while keeping VITVO outside the incubator at 37°C.

Supplementary Figure 3

RNA extraction from VITVO 3D culture and Real Time PCR (a) Total RNA was isolated using Trizol directly in VITVO and used for quantitative real-time PCR (qRT-PCR). **(b)** Comparison between Ct values obtained from qRT-PCR reaction using beta actin (B-ACT) and GAPDH primers and starting from 2D- and 3D-cultures RNA.

Supplementary Figure 4

In VITVO evaluation of two different concentration of Real Time-Glo reagent. A673 were seeded in two VITVO devices at the same density (560.000/device) and Real Time-Glo was added every 24 hours at 1X or 2X final concentration, comparing the measured RLU.

Supplementary Figure 1

a

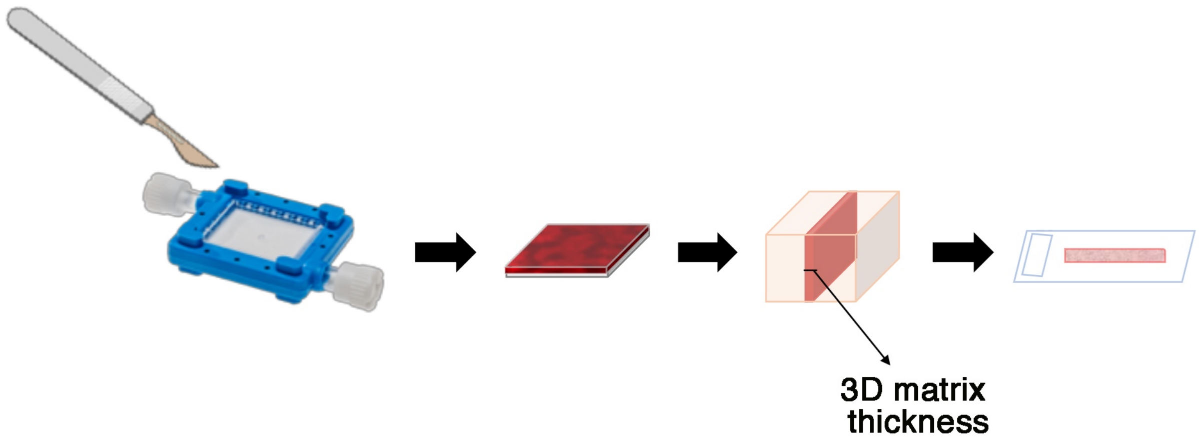

b

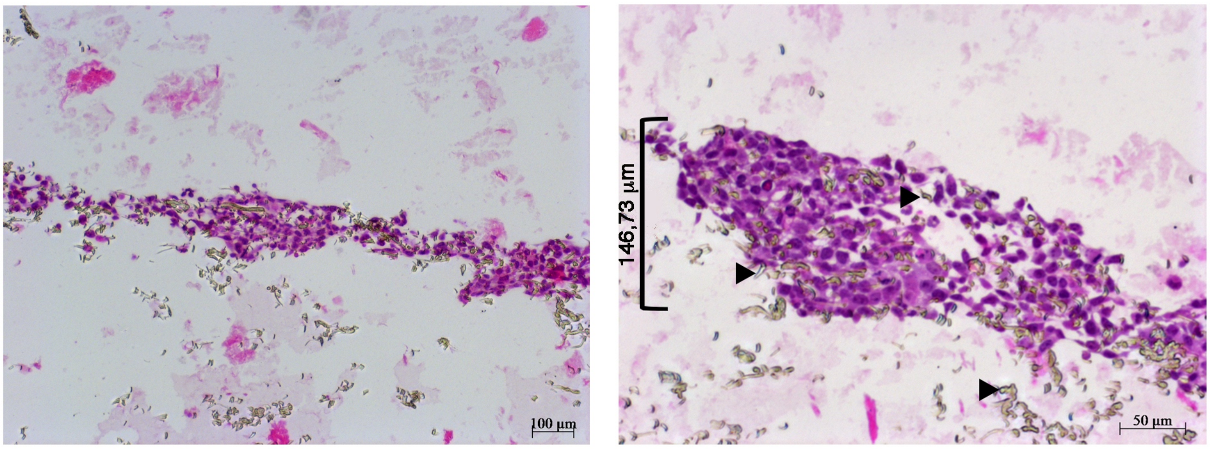

Supplementary Figure 2

a

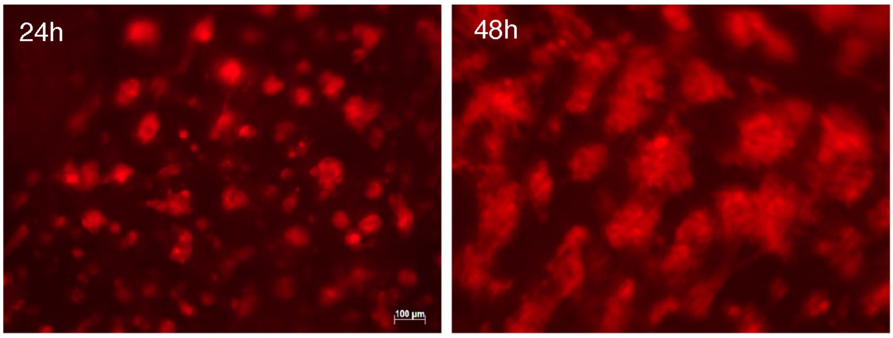

b

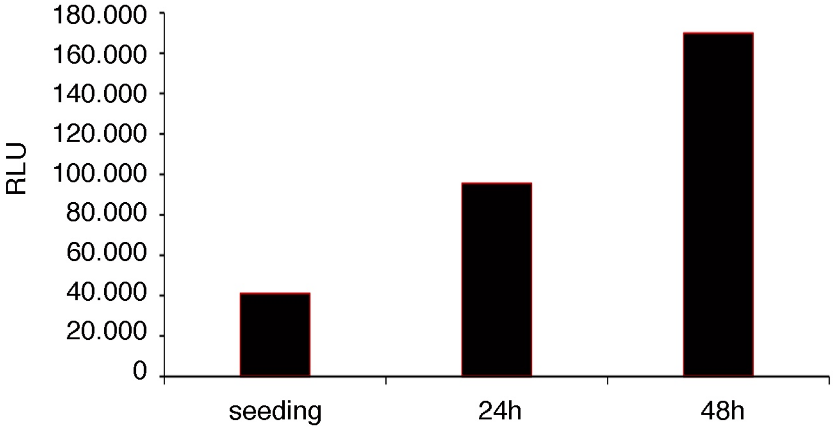

Number of cells	560.000 (inoculum)	1.302.400 (estimated)	2.316.900 (estimated)

Supplementary Figure 3

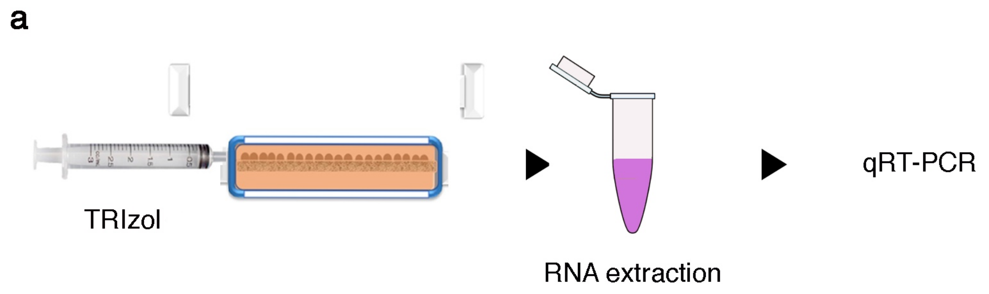

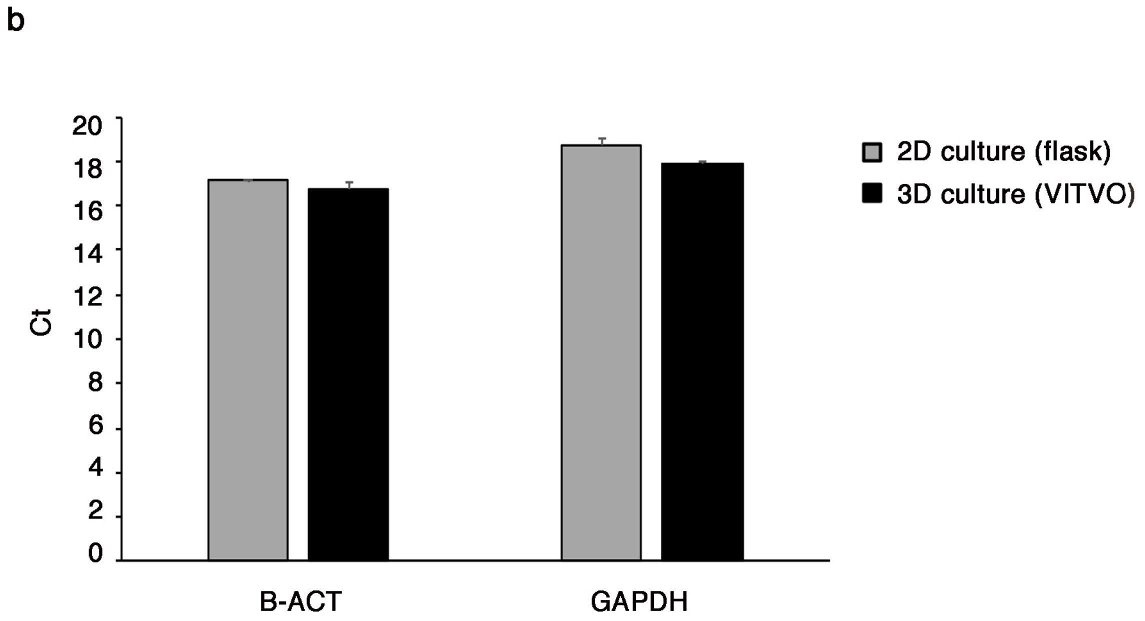

Supplementary Figure 4

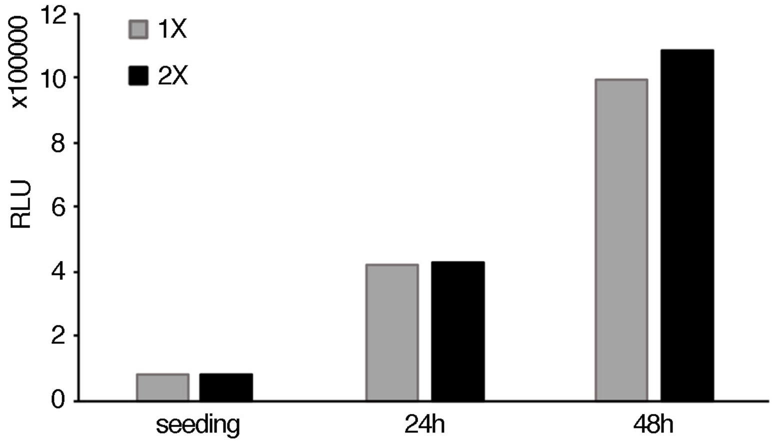

Supplement: Supplementary file 2 — Supplementary Information [file 41598_2019_43613_MOESM2_ESM.pdf]
